# *KCNG1*-Related Syndromic Form of Congenital Neuromuscular Channelopathy in a Crossbred Calf

**DOI:** 10.3390/genes12111792

**Published:** 2021-11-12

**Authors:** Joana G. P. Jacinto, Irene M. Häfliger, Eylem Emek Akyürek, Roberta Sacchetto, Cinzia Benazzi, Arcangelo Gentile, Cord Drögemüller

**Affiliations:** 1 Department of Veterinary Medical Sciences, University of Bologna, 40064 Ozzano Emilia, Italy; joana.goncalves2@studio.unibo.it (J.G.P.J.); cinzia.benazzi@unibo.it (C.B.); arcangelo.gentile@unibo.it (A.G.); 2 Institute of Genetics, Vetsuisse Faculty, University of Bern, 3012 Bern, Switzerland; irene.haefliger@vetsuisse.unibe.ch; 3 Department of Comparative Biomedicine and Food Science, University of Padova, 35020 Legnaro, Italy; eylememek.akyurek@phd.unipd.it (E.E.A.); roberta.sacchetto@unipd.it (R.S.)

**Keywords:** cattle, channelopathy, skeletal muscle, neuromuscular disorder, paradoxical myotonia congenita, potassium voltage-gated channel, precision medicine, hydrosyringomyelia, craniofacial dysmorphism

## Abstract

Inherited channelopathies are a clinically and heritably heterogeneous group of disorders that result from ion channel dysfunction. The aim of this study was to characterize the clinicopathologic features of a Belgian Blue x Holstein crossbred calf with paradoxical myotonia congenita, craniofacial dysmorphism, and myelodysplasia, and to identify the most likely genetic etiology. The calf displayed episodes of exercise-induced generalized myotonic muscle stiffness accompanied by increase in serum potassium. It also showed slight flattening of the splanchnocranium with deviation to the right side. On gross pathology, myelodysplasia (hydrosyringomielia and segmental hypoplasia) in the lumbosacral intumescence region was noticed. Histopathology of the muscle profile revealed loss of the main shape in 5.3% of muscle fibers. Whole-genome sequencing revealed a heterozygous missense variant in *KCNG1* affecting an evolutionary conserved residue (p.Trp416Cys). The mutation was predicted to be deleterious and to alter the pore helix of the ion transport domain of the transmembrane protein. The identified variant was present only in the affected calf and not seen in more than 5200 other sequenced bovine genomes. We speculate that the mutation occurred either as a parental germline mutation or post-zygotically in the developing embryo. This study implicates an important role for *KCNG1* as a member of the potassium voltage-gated channel group in neurodegeneration. Providing the first possible *KCNG1*-related disease model, we have, therefore, identified a new potential candidate for related conditions both in animals and in humans. This study illustrates the enormous potential of phenotypically well-studied spontaneous mutants in domestic animals to provide new insights into the function of individual genes.

## 1. Introduction

Inherited channelopathies represent a clinically and heritably heterogeneous group of genetic disorders that result from a ion channel dysfunction of all cellular plasma membranes and/or of cell organelles [1]. They usually follow a dominant inheritance [1].

Neuromuscular channelopathies can cause different diseases affecting the brain, spinal cord, peripheral nerve, and/or muscle [2]. In particular, those that lead to primary skeletal muscle diseases, the so-called skeletal muscle channelopathies, exhibit a clinical spectrum ranging from flaccid paralysis to myotonia, the latter defined as delayed relaxation of a muscle that has been voluntarily or reflexively contracted [3].

In human medicine, skeletal muscle channelopathies are associated with pathogenic variants in genes coding for ion channels that influence muscle excitability [1,4]. They are subdivided into two types, periodic paralysis (PPs) and non-dystrophic myotonias (NDMs) [5].

Various forms of PPs are due to abnormal depolarization that inactivates sodium channels, causing decreased muscle excitability of the muscle membrane, often accompanied by changes in extracellular potassium. The main finding is the susceptibility to episodes of focal or generalized weakness and paralysis [1]. This group of channelopathies includes three conditions: (a) hypokalemic periodic paralysis (HypoPP); (b) hyperkalemic periodic paralysis (HyperPP); and (c) Andersen-Tawil syndrome. HypoPP is the most common PP in humans, characterized by episodes of flaccid muscle weakness associated with low serum potassium levels, lasting hours to days [6]. The attacks of flaccid paralysis typically occur after waking during the night or early morning. It is due to a dysfunction of calcium channels associated with heterozygous variants in *CACNA1S* [7] or to a dysfunction of sodium channels associated with heterozygous variants in *SCN4A* [8]. HyperPP is characterized by episodes of flaccid muscle weakness associated with elevated serum potassium levels and occasional myotonia lasting minutes to hours [2]. It is due to dysfunction of sodium channels also associated with mutations in *SCN4A* [9]. The Andersen–Tawil syndrome is characterized by the following clinical triad: periodic paralysis, cardiac manifestations, and abnormal physical features. It is due to dysfunction of potassium channels associated with variants in *KCNJ2* [2,10]. In veterinary medicine, a *SCN4A*-related equine form of HyperPP has been reported (OMIA 000785-9796) [11].

Various forms of NDMs are due to defects in the muscle fiber repolarization, resulting in muscle hyperexcitability and myotonic discharges [10]. The main findings are muscular stiffness, in the absence of severe fixed weakness or muscle wasting, and muscle hypertrophy [4]. NDMs encompasses three different disorders, defined as following: (a) myotonia congenita (MC); (b) paramyotonia congenita (PMC); and (c) Na channel myotonias [6]. MC is the most common skeletal muscle channelopathy in humans, characterized by stiffness especially during rapid movements after a period of rest (action myotonia), and improving with exercise (“warm-up phenomenon”) [3]. It is due to a dysfunction of chloride channels associated with dominant (Thomsen’s disease) or recessively (Becker’s disease) inherited mutations in *CLCN1* [12]. PMC is characterized by stiffness that, unlike MC, worsens with sustained exercise (exercise-induced or paradoxical myotonia) [6]. The symptoms last for seconds to minutes following the exercise. It is due to a dysfunction of sodium channels [13] also associated with heterozygous pathogenic variants in *SCN4A* [14]. There are several subtypes of sodium channel myotonia, such as acetazolamide-responsive myotonia, myotonia that develops approximately 10–20 min after exercise (*myotonia fluctans)* [15], and severe persistent myotonia associated with unique electromyographic pattern (*myotonia permanens)* [16]. Common to these subtypes is exacerbation by K (potassium-aggravated myotonias). Similarly to the PMC, they are all also associated with heterozygous pathogenic variants in *SCN4A* [14].

In veterinary medicine, forms of MC have been reported in horses (OMIA 000698-9796) [17], dogs (OMIA 000698-9615) [18], cats (OMIA 000698-9685) [19], sheep (OMIA 000698-9940) [20], and goats (OMIA 000698-9925) [21], associated with pathogenic variants in the orthologue *CLCN1* genes.

Altogether, it can be said that many clinicopathological similarities to *CLCN1-* and *SCN4A*-related human genetic diseases can be evidenced in veterinary pathology, highlighting the usefulness of translational research in the field of the congenital neuromuscular channelopathies. To our knowledge, no neuromuscular channelopathies have been reported in cattle. Therefore, with the present study we intended to characterize the clinical and pathological phenotype of a crossbred calf affected by congenital paradoxical myotonia, craniofacial dysmorphism, and myelodysplasia, and to find a possible genetic explanation after whole-genome sequencing (WGS).

## 2. Materials and Methods

### 2.1. Clinical and Pathological Investigation

A five-day-old male Belgian Blue x Holstein crossbred calf, weighting 47 kg, was admitted to the University of Bologna due to difficulty on quadrupedal stance and locomotion due to generalized muscle stiffness present since birth. The affected calf was clinically examined and a complete blood count (CBC), serum biochemical analysis, and venous blood gas analysis were obtained. Blood gas and serum biochemical analysis were performed at rest and after stimulation. Stimulation was the term used when the calf was in quadrupedal stance.

Nineteen days after hospitalization the calf showed a worsening of the general condition related to neuromuscular disease and was euthanized for welfare reasons. The calf was subsequently submitted for necropsy and histologic examination. Semimembranosus muscle was fixed in buffered neutral paraformaldehyde at 4 °C, washed in phosphate-buffered saline and de-hydrated through a graded series of ethanol. Samples embedded in paraffin were cut at 5 µm and stained with hematoxylin and eosin (H&E), or Azan–Mallory method, specific for detection of collagen fibers. Muscle sections were scanned with a semiautomatic microscope equipped (D-Sight v2, Menarini Diagnostics, Florence, Italy) with a computer. The average percentage of pathological muscle fibers was determined as the ratio of muscle fibers that lost their main shape and/or took a round shape to the total muscle fibers in the region. The spinal cord was fixed in 10% buffered formalin, embedded in paraffin, cut at 4 µm, and stained with hematoxylin and eosin (H&E), Periodic acid-Schiff (PAS), and Luxol-Fast-Blue for histological evaluation.

### 2.2. DNA Extraction, Whole-Genome Sequencing and Variant Calling

Genomic DNA was isolated from EDTA blood of the affected calf using a Promega Maxwell RSC DNA system (Promega, Dübendorf, Switzerland). Using genomic DNA from the affected calf, an individual PCR-free fragment library with approximately 400 bp inserts was created and sequenced on a NovaSeq6000 for 150 bp paired-end reads (Illumina, San Diego, CA, USA). The sequenced reads were aligned to the ARS-UCD1.2 reference genome, resulting in an average coverage of approximately 17.4× [22], and single-nucleotide variants (SNVs) and small indel variants were called. The applied software and steps to process fastq files into binary alignment map (BAM) and genomic variant call format (GVCF) files were in accordance with the processing guidelines of the 1000 Bull Genomes Project (run 7) [23], with the exception of trimming, which was performed with fastp [24]. Further processing of the genomic data was performed according to Häfliger et al. 2020 [25]. The effects of the above variants were functionally evaluated with snpeff v4.3 [26], using the NCBI Annotation Release 106 (https://www.ncbi.nlm.nih.gov/genome/annotation_euk/Bos_taurus/106/; accessed on 17 July 2021). This resulted in the final VCF file, containing individual variants and their functional annotations. To find private variants, we compared the genotypes of the case with 691 cattle genomes of different breeds sequenced as part of the ongoing Swiss Comparative Bovine Resequencing project. All of its data are available (Table S1; https://www.ebi.ac.uk/ena/browser/view/PRJEB18113 accessed on 17 July 2021) in the European Nucleotide Archive (SAMEA7690196 is the sample accession number of the affected calf). Integrative Genomics Viewer (IGV) [27] software version 2.0 was used for visual evaluation of genome regions containing potential candidate genes.

### 2.3. Validation and Selection of Potential Canidate Variants

#### 2.3.1. Occurrence of Variants in a Global Control Cohort

The comprehensive variant catalogue of run 9 of the 1000 Bull Genomes Project was available to investigate the allele distribution of variants within a global control cohort (www.1000bullgenomes.com; accessed on 17 July 2021) [23]. The whole data set includes 5116 cattle genomes including 576 from the Swiss Comparative Bovine Resequencing project, from a variety of breeds (>130 breeds indicated). Within the dataset, there are 9 purebred Belgian Blue and 1209 purebred Holstein cattle, allowing for the exclusion of common variants.

#### 2.3.2. In Silico Assessment of the Molecular Consequences of Amino Acid Exchanges

Mutpred2 [28], PROVEAN [29] and PredictSNP1 [30] were used to predict the biological consequences of the detected missense variant. For cross-species sequence alignments, the following NCBI protein accessions were considered: NP_001192648.1 (*Bos taurus*), NP_002228.2 (*Homo sapiens*), XP_001168521.2 (*Pan troglodytes*), XP_543053.2 (*Canis lupus*), NP_001074603.1 (*Mus musculus*), NP_001100015.1 (*Rattus norvegicus*), XP_004947317.1 (*Gallus gallus*), and NP_001103880.1 (*Danio rerio*), NP_001096675.1 (*Xenopus tropicalis*).

### 2.4. Sequence Accessions

All references to the bovine *KCNG1* gene correspond to the NCBI accessions NC_037340.1 (chromosome 13, ARS-UCD1.2), NM_001205719.1 (*KCNG1* mRNA), and NP_001192648.1 (KCNG1 protein). For the protein structure of KCNG1 the Uniprot database (https://www.uniprot.org/; accessed on 17 July 2021) with accession number Q9UIX4 was used.

## 3. Results

### 3.1. Clinical Phenotype

On clinical examination at the time of admission, the calf was bright and alert but with generalized muscle stiffness that prevented it from spontaneously assuming and maintaining the quadrupedal stance.

**Figure 1:**
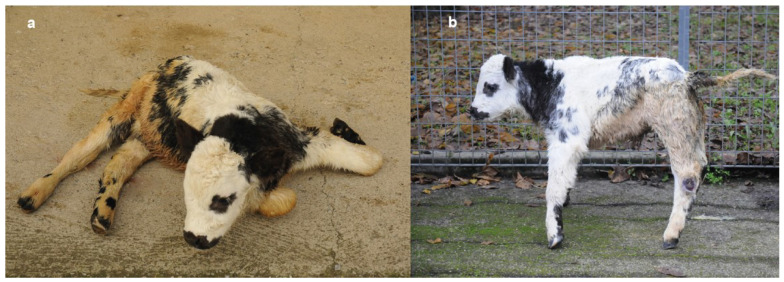
Crossbred calf with a congenital neuromuscular disorder characterized by paradoxical myotonia congenita and myelodisplasia. (**a**) Sternal recumbency at rest. Note that the calf sustains the forelimbs folded underneath its chest while the hindlimbs are hyperextended. (**b**) Quadrupedal stance after passive positioning. Note that the pelvic girdle appears to be more affected with a marked hyperextension of the hindlimbs, the back is slightly arched, and the tail head is elevated. The thoracic girdle was affected with the forelimbs’ hoofs resting on tip.

At rest, the animal preferred the sternal recumbency, with the forelimbs folded under its chest while the hindlimbs were rigid and hyperextended (Figure 1a). It was not possible to flex the hindlimbs due to the muscle stiffness. If stimulated to stand the muscle stiffness increased inducing a rigid posture accompanied by spastic contractions that prevented him to acquire a definitive quadrupedal stance. On the contrary, if gently passively positioned, the calf was able to acquire and maintain the quadrupedal stance. In standing, the hind limbs were contracted and hyperextended, especially the right hindlimb that showed caudal stretching (Video S1). Additionally, the back was slightly arched, and the tail head elevated (Figure 1b). The thoracic girdle was also involved but less severely. On hooping and hoof replacement, the calf was unable to re-acquire the physiological position of the limbs/hoof. Unless supported, the calf was unable to walk or maintain the quadrupedal stance for long time. In fact, uncontrolled hypertonic postural reactions and muscular contractions resulted in loss of stance, with a fall in lateral recumbency. If not further stimulated and stressed, the stiffness slowly tended to decrease, enabling the calf to acquire the sternal recumbency. However, the hypertonia never disappeared completely. The cutaneous trunci reflex was increased in intensity as well as the withdrawal reflex of the forelimbs while in the hindlimbs the latter was absent. No abnormalities in the cranial nerves’ reflexes, threat response, and pain perception were noticed.

The calf showed a slight flattening of the splanchnocranium with deviation to the right side. It displayed carpal and tarsal skin lesions due to permanent recumbency. Moreover, the animal presented diarrhea.

CBC revealed moderate leukocytosis (23,650/mm^3^) with neutrophilia (14,280/mm^3^) and monocytosis (2400/mm^3^). Serum biochemical profile after stimulation showed increase in: creatinine kinase, lactate dehydrogenase (LDH), L-lactate, potassium (K^+^), and calcium (Ca^2+^) (Table 1).

Based on the clinical findings, the calf was suspected of suffering from a form of paradoxical myotonia congenita and/or from a spinal cord lesion associated with craniofacial dysmorphism.

### 3.2. Pathological Phenotype

**Figure 2:**
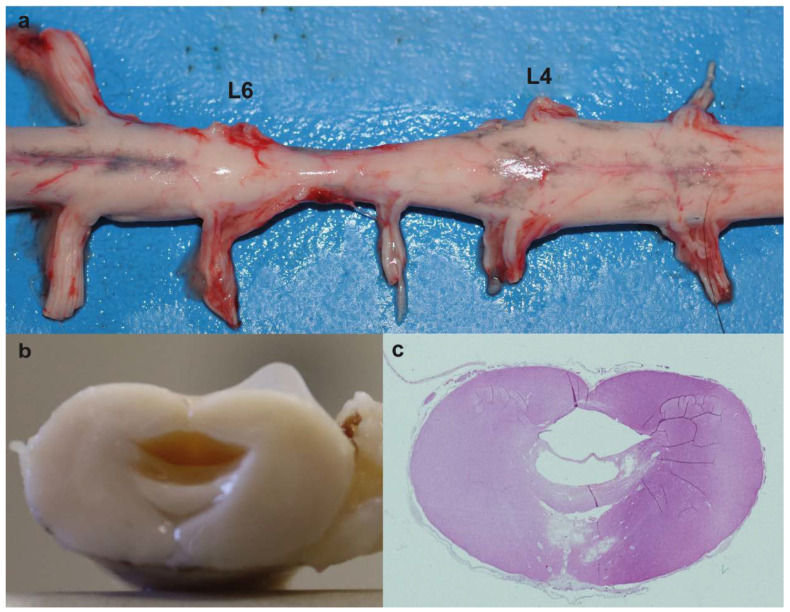
Myelodysplasia associated with hydrosyringomyelia in the affected calf. (**a**) Note the narrowing of the spinal cord between lumbar spinal nerves IV (L4) and VI (L6) (myelodysplasia). (**b**) Transversal section of the spinal cord between lumbar spinal nerve V (L5) and VI (L6). Note the cavity formed within the spinal cord. (**c**) Histological section of (**b**). Note that there are two cavities with only the larger partially lined by ependymal cells (hydrosyringomielia). hematoxylin and eosin (H&E) staining.

At gross pathology, the examination of central nervous system revealed narrowing of the spinal cord (myelodysplasia) between lumbar spinal nerves IV and VI associated with hydrosyringomyelia between lumbar spinal nerves III and V with the larger cavity at the level of lumbar spinal nerve IV (Figure 2a,b). Macroscopically, the muscles were normal.

Microscopically, at lumbar spinal nerves III to V there were two cavities with only the larger partially lined by ependymal cells (hydrosyringomielia) (Figure 2c). Vasogenic and intramyelinic edemas were present in the areas around the cavities, characterized as multiple fluid-filled clear extracellular spaces in the gray matter. Around the capillaries near the smaller channel, there was neutrophilic and lymphoid inflammatory cells. The astrocytes in the white matter showed foci of chromatin margination.

**Figure 3:**
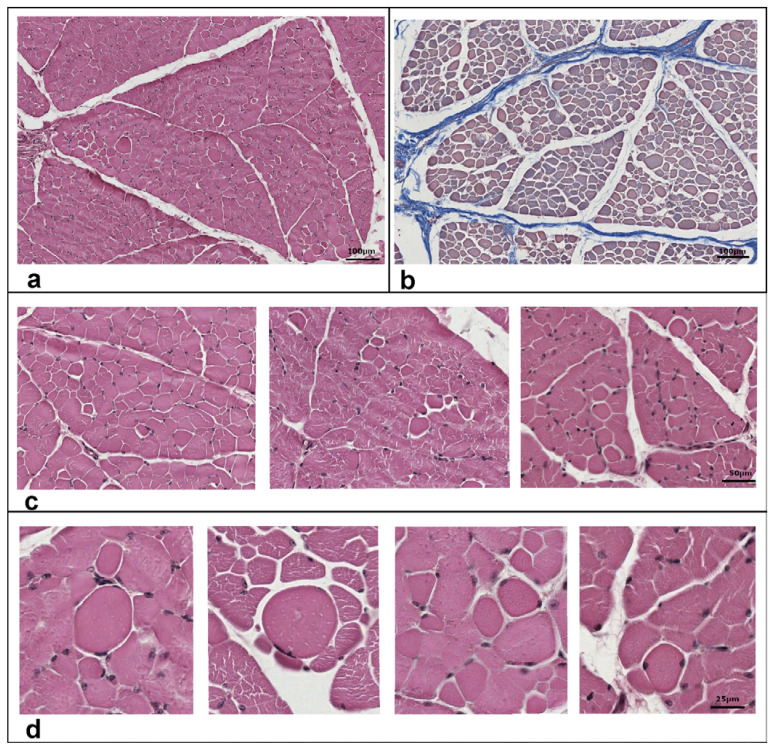
Histological features of semimembranosus muscle of the studied case. Transversal sections from muscle biopsies were stained with hematoxylin and eosin (**a**,**c**,**d**) or with Azan–Mallory method (**b**) to identify collagen fibers. Enlarged round shaped fibers are highlighted in panels (**c**,**d**). The percentage value of round shaped fibers (5.3%) was determined as the ratio of muscle fibers that lost their main shape and/or took a round shape to the total muscle fibers in the region. Scale bars correspond to 100 m in panels (**a**,**b**), and 50 and 25 m in panels c and d, respectively.

Routinely morphological (hematoxylin-eosin) analysis was used for histopathological evaluation on semimembranosus muscle biopsy sections. Muscle parenchyma showed normal fibers distribution. (Figure 3a). Nevertheless, some fibers appeared round shaped (Figure 3c) and most of them exhibited an enlarged cross-sectional area (Figure 3d). It was determined that the average percentage of pathological muscle fibers was 5.3%. A possible presence of fibrosis was determined by Azan–Mallory staining (Figure 3b). No signs of fibrosis were found in the sections obtained from the tissue. Infiltrated inflammatory mononuclear cells were not revealed by histological analysis of the muscle.

### 3.3. Genetic Analysis

Assuming spontaneous mutation as the cause of this congenital neuromuscular condition, the WGS data were filtered for heterozygous coding variants that were present in the calf and were absent in the 691 available cattle genomes of different breeds. Thereby, 151 variants with a predicted high or moderate impact were identified (Table 2). In a second step, these variants were analyzed for their occurrence in a global cohort of 4540 genomes from a variety of breeds. This revealed 27 remaining protein-changing variants that are exclusively heterozygous in the affected calf and absent in all controls. These 27 variants were then visually inspected using the IGV software (Broad institute, Cambridge, MA, USA), which confirmed 25 as true variants (Table 2; Table S2).

**Figure 4:**
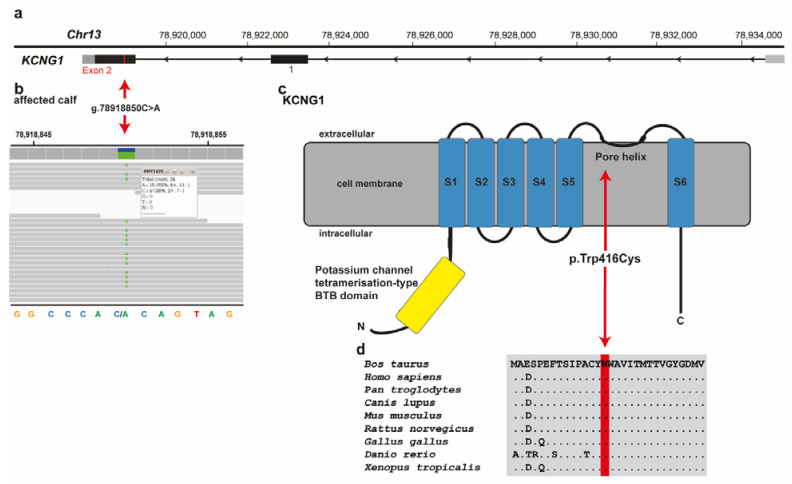
*KCNG1* missense variant in a crossbred calf with paradoxical myotonia congenita and myelodysplasia. (**a**) Structure of *KCNG1* showing the exon 2 variant located on chromosome 13. (**b**) IGV screenshot presenting the Chr13: 78918850C>A variant in the affected calf. (**c**) Schematic representation of *KCNG1* protein and its functional domains with the position of the identified pathogenic variant (red arrow). The six transmembrane domains are shown in blue (S1-S6). (**d**) Cross-species sequence comparison of the ion transport domain of the KCNG1protein with the region around the p.Trp416Cys variant shows complete evolutionary conservation.

Among these 25 remaining private variants, one single variant affects an interesting functional candidate gene for the studied phenotype (Figure 4a). This heterozygous variant at chr13:78918850C>A is a missense variant in exon 2 of the *potassium voltage-gated channel modifier subfamily G member 1* (*KCNG1*) gene (NM_001205719.1: c.1248G>T; Figure 4b). It exchanges the encoded amino acid of *KCNG1* at position 416 (NP_001192648.1: p.Trp416Cys), located in the pore helix of the ion transport domain (Figure 4c). Furthermore, the tryptophan-to-cysteine substitution affects a highly conserved residue (4d), and was predicted to be deleterious by three different tools (Mutpred2 score 0.951; PROVEAN score -12.212; PredictSNP1 score 0.869) and to alter the transmembrane protein and ordered interface. Unfortunately, biological samples of the dam and sire that were slaughtered in the meantime, were not available. Analysis of the other 24 identified variants, taking into account the known function of the gene, the reported association with Mendelian diseases, and/or the in silico assessment of the molecular consequences of the variants in the protein, did not reveal any other plausible cause for the observed phenotype (Table S2).

Assuming recessive inheritance, filtering of WGS data for homozygous coding variants present in the calf and missing in the 691 control genomes of different breeds identified 12 variants with likely moderate impact. These 12 variants were further investigated for their occurrence in a diverse cohort of additional 4540 bovine genomes, which revealed the absence of variants only present homozygous in the affected calf (Table 2).

## 4. Discussion

This study aimed to investigate the clinicopathological phenotype and the underlying genetic cause in a crossbred calf displaying paradoxical myotonia congenita, craniofacial dysmorphism and myelodysplasia. The phenotype of this syndromic form of congenital neuromuscular disorder displays striking similarities with *SCN4A*-related forms of human PMC because it presents with episodes of exercise-induced generalized myotonic muscle stiffness. Clinical diagnosis of PMC in humans is based on consistent history, and typical clinical and electromyographic findings [6].

In PMC patients, changes in muscle fiber diameters and internal nuclei are also among the nonspecific histologic features suggestive of mild myopathic changes [31]. The muscle profile in this study revealed that 5.3% of muscle fibers lost their main shape and/or took a round shape to the total muscle fibers in the region, findings that are consistent with the human PMC features. However, the lack of an electromyography prevented a definite categorization of this paradoxical myotonia as PMC.

Moreover, some further phenotypical differences from the typical form of human PMC were noticed, such as: (1) increase in serum K^+^ after stimulation; (2) inability to relax muscle immediately after the stimulation; (3) craniofacial dysmorphism; and (4) permanent extension of the hindlimbs, the latter explained by the retrieved myelodysplasia associated with hydrosyringomyelia in the lumbosacral intumescence region. Taken together, the muscle stiffness episodes and the findings of points (1) and (2) more resembled *CACNA1S*-related forms of HyperPP. Human patients with this genetic disease show an increase in serum potassium during an attack [4]. Moreover, approximately half of the patients display muscle stiffness arising from myotonia or paramyotonia [32]. On the other side, considering the clinical muscle findings and point (3)—craniofacial dysmorphism—the observed phenotype shows similarities to the human *KCNJ2*-related Andersen–Tawil syndrome.

Hence, to the best of our understanding, our patient showed a previously unreported combination of paradoxical myotonia congenita, hyperkalemia during episodes, craniofacial dysmorphism, and myelodysplasia associated with hydrosyringomyelia, representing a novel clinicopathological presentation.

In humans, genetic confirmation of known pathogenic variants in *SCN4A*-related PMC and HyperPP, and *KCNJ2*-related Andersen–Tawil syndrome-related, is included in the diagnosis of these disorders [6]. In the studied calf, no candidate causal variant in *CACNA1S, SCN4A*, or *KCNJ2* were found by genome re-sequencing. Likewise, in human medicine, patients with phenotypical characteristics of PMC, HyperPP, and Andersen–Tawil syndrome were found not to present pathogenic variants in the *SCN4A* or in *KCNJ2*, suggesting further genetic heterogeneity [33]. Therefore, we evaluated the possible genetic cause for this novel congenital phenotype systematically, assuming both recessively inherited and de novo mutations. Our results from the analysis of WGS data showed that there was not a single homozygous protein-changing variant present in the affected calf, ruling out a possible recessive inheritance as the most likely cause. Furthermore, as our case was an offspring of a crossbred mating it seems to be highly unlikely that a monogenic recessive variant was causal. Especially since no bovine simple genetic disease is known that segregates in such diverse dairy and beef breeds as Holstein and Belgian-Blue, respectively. Therefore, the more plausible explanation would be to search for allelic heterogeneity, meaning two different (breed-specific) coding variants affecting the same gene. Our results from the WGS identified 25 heterozygous private protein-changing variants present in the genome of the affected calf which were absent in a cohort of more than 5200 cattle genomes. Considering the known function of the affected gene, the rarity of the variant, and the outcome of the in silico effect prediction, the identified heterozygous *KCNG1* missense variant was assumed to represent the most likely genetic cause for the observed phenotype. We could only speculate that the mutation either occurred post-zygotically in the developing embryo or it represents a germline mutation in the dam or sire. To confirm that the identified mutation in the *KCNG1* gene occurred indeed de novo, genotyping of the parents would be needed. Unfortunately, no genetic material of both parents was available. To the best of our knowledge, no pathogenic variant in *KCNG1* gene has been reported in animals and humans. Therefore, this study represents the first example of a *KCNG1*-related neuromuscular disorder as a conserved residue in the pore helix of the ion transport domain of the potassium voltage-gated channel subfamily G member 1 protein is altered.

Voltage-gated potassium channels represent a family of transmembrane proteins that are highly expressed in the central nervous system of the mammalian species, playing a major role in the control of neuronal excitability [34]. Additionally, they regulate a variety of electrophysiological properties, including the interspike membrane, the wave-form of the action potential and the firing frequency [35]. In particular, *KCNG1* is a potassium channel subunit that cannot form functional channels by itself [36]. However, it forms functional channels with the *KCNB1* (one of the most important voltage-gated potassium channels) and modulates the delayer rectifier voltage-gated potassium channel activation and deactivation rated of this protein [34,37]. Specifically, *KCNG1* in co-expression with *KCNB1* results in potassium channels that have slower kinetics of deactivation, due to a negative shift of the steady-state activation curve, and marked slowing of deactivation tail currents [34,37]. The performed in silico evaluations of the identified p.Trp416Cys mutation predicted that this mutation altered the transmembrane protein and ordered interface. Therefore, we hypothesize that our mutant protein leads to a malfunction of the encoded channels allowing an additional release of intracellular potassium from the skeletal muscle. Subsequently, this change in ion transport impairs the ability of the muscle to contract, leading to the observed stiffness.

Moreover, in human medicine, there are several potassium channelopathies whose presentations are suggestive of developmental disorders, with findings including intellectual disability, craniofacial dysmorphism or other physical abnormalities [38]. Physiological functions of *KCNG1* are largely unknown, by similarity, therefore, it is plausible that the identified mutation in our study is also the underlying cause for the craniofacial dysmorphism and myelodysplasia associated with hydrosyringomyelia.

## 5. Conclusions

We have uncovered a novel phenotype of a most likely dominantly inherited neuromuscular channelopathy in cattle related to a potentially pathogenic variant in the bovine *KCNG1* gene. Targeted expression of affected potassium channels in transfected cell lines, in combination with recently developed gene editing tools such as CRISPR/Cas9 to mimic the consequences of the exchanged residue, may be suitable to functionally prove our claims in the future. Nevertheless, we propose here the first *KCNG1* mutation present with a disorder in a mammalian species. Our study highlights that the genetics of inherited disorders in well-phenotyped large animals, such as cattle, is a valuable model system for studying fundamental aspects of gene function.

## Figures and Tables

**Table 1:** Results of altered parameters of serum biochemical profiles of the affected calf at rest and after stimulation.

Parameter	At Rest	After Stimulation	Unit of Measure
Creatinine kinase (CK)	147	329	IU/L
Lactate dehydrogenase (LDH)	1975	2556	IU/L
L-lactate	1	3.8	mmol/L
Potassium (K+)	3.6	4.3	mmol/L
Calcium (Ca2+)	0.99	1.14	mmol/L

**Table 2:** Results of whole-genome sequencing variant filtering of the calf affected by paramyotonia congenita and myelodysplasia.

Filtering Step	Homozygous Variants	Heterozygous Variants
All variants	2,562,043	5,168,233
Private variants	3580	21,104
Protein-changing private variants using 691 cattle genome controls	12	115
Remaining protein-changing private variants using a global control cohort of 4540 cattle genomes and subsequent IGV inspection	0	25

## Data Availability

The whole-genome data of the affected calf (sample ID PMT1625) is freely available at the European Nucleotide Archive (ENA) under sample accession number SAMEA7690196.

## Supplementary Materials

The following are available online at https://www.mdpi.com/article/10.3390/genes12111792/s1, Table S1: EBI Accession numbers of all publicly available genome sequences. We compared the genotypes of the calf with 691 cattle genomes of various breeds that had been sequenced in the course of other ongoing studies and that were publicly available; Table S2: List of the remaining variants after the comparison to the global control cohort of 5116 genomes of other breeds (1000 Bull Genomes Project run 9; www.1000bullgenomes.com; acceded on 17 July 2021) and after IGV visual inspection, revealing 25 protein-changing variants with a predicted moderate or high impact only present in the affected calf; Video S1: After passively positioned, the calf was able to acquire and maintain the quadrupedal stance. In standing the hind limbs were contracted and hyperextended, and tail head elevated.
